# Comparison between optical and digital blur using near visual acuity

**DOI:** 10.1038/s41598-021-82965-z

**Published:** 2021-02-09

**Authors:** David Kordek, Laura K. Young, Jan Kremláček

**Affiliations:** 1 Department of Medical Biophysics, Faculty of Medicine in Hradec Kralove, Charles University, Hradec Kralove, Czech Republic; 2 Biosciences Institute, Faculty of Medical Sciences, Newcastle University, Newcastle, UK; 3 Department of Experimental Psychology, University of Oxford, Oxford, UK; 4 Department of Pathological Physiology, Faculty of Medicine in Hradec Kralove, Charles University, Hradec Kralove, Czech Republic

**Keywords:** Sensory processing, Imaging and sensing

## Abstract

In a low-cost laboratory setup, we compared visual acuity (VA) for stimuli rendered with Zernike aberrations to an equivalent optical dioptric defocus in emmetropic individuals using a relatively short observing distance of 60 cm. The equivalent spherical refractive error of + 1, + 2 or + 4 D, was applied in the rendering of Landolt Rings. Separately, the refractive error was introduced dioptrically in: (1) unchanged Landolt Rings with an added external lens (+ 1, + 2 or + 4 D) at the subject's eye; (2) same as (1) but with an added accommodation and a vertex distance adjustment. To compare all three approaches, we examined VA in 10 healthy men. Stimuli were observed on a PC CRT screen. For all three levels of refractive error, the pairwise comparison did not show a statistically significant difference between digital blur and accommodation-plus-vertex-distance-adjusted dioptric blur (p < 0.204). The best agreement, determined by Bland–Altman analysis, was measured for + 4 D and was in line with test–retest limits for examination in the clinical population. Our results show that even for a near observing distance, it is possible to use digitally rendered defocus to replicate dioptric blur without a significant change in VA in emmetropic subjects.

## Introduction

The aim of our study was to design an image modification procedure to simulate visual defocus. We were interested in an approach, sometimes called a source modification, which transforms an original image at the level of generation (e.g. when rendered on a computer screen)—i.e. digital blur. As an alternative approach, a visual image can be degraded at the position of an observer, e.g. using lenses—i.e. dioptric blur. There are alternative and more sophisticated methods to generate the defocus at the observer side, such as using a deformable mirror or liquid crystal spatial light modulator in an adaptive optics system^1,2^. These systems are complex, but a commercial device exists (VAO, Voptica). While more expensive, such systems have the ability to manipulating higher-order as well as lower-order aberrations. The advantage of our digital approach is an easy implementation using a standard office PC. Moreover, digital blur brings several advantages over the dioptric approach, including a low sensitivity to eyelid squinting, removal of optical setup limitations, such as the variability of the lens-to-eye distance and alignment, and overcoming the restricted set of lens powers available.

A number of authors have described the use of simulation for predicting an individual’s visual acuity (VA) from a wavefront measurement. These include a model of visual processing to estimate visual acuity based on image quality and they show good agreement with the corresponding clinical acuity measurements^3–7^. Here, instead, we ask whether digital blurring results in the same VA as dioptric blurring by the same amount. Smith et al*.* compared VA measured using source (digital) versus observer (dioptric) blur and found a high correlation between the two methods, although digital blur resulted in a lower VA, which was statistically, but not clinically, significant^8^. Ohlendorf et al*.* used a similar approach to compare VA measured using real and simulated refractive errors, including spherical refractive error and astigmatism, and found that there was a high correlation between VA measured by the two methods^9^. They also found a slight tendency for the same VA to result from a smaller amount of simulated defocus than optical defocus, although this only reached significance for one amplitude of defocus that was tested. Similarly, Remón et al. found no significant difference between digital and dioptric blur, with a tendency for digital blur to result in a slightly lower VA for all but one participant^10^. Dehnert et al*.* found comparable measurements of VA for digital and dioptric blur but, on the contrary, with a tendency for dioptric blur to result in slightly lower VA^11^. None of these studies found a clinically significant difference between the two methods, suggesting that digital and dioptric blur can be used interchangeably for clinical measures of VA.

Contrary to other experiments of this kind we intended to test the digital blur in a short observing distance (e.g. 60 cm), which is frequently used in computer-related vision tests e.g. visual evoked potentials^12^ or the electroretinogram^13^. Such examinations require precise timing, and the monitor spatial resolution is often lower than is necessary for a VA examination. From this concept a specific question arose—can a relatively low monitor resolution and short observing distance provide comparable effects for rendered and dioptric defocus? For the purposes of this study, 60 cm was chosen as a suitable viewing distance to comply with the International Society for Clinical Electrophysiology of Vision standard^12^ while also allowing for a large stimulating field.

In our study we simulated spherical refractive errors using a Fourier optical technique implemented in the Python programming language (Python Software Foundation, https://www.python.org/) using the Numpy library^14^. We measured VA in emmetropic individuals, quantified by the logarithm of the minimum angle of resolution, logMAR $= {\text{log}}\;1/VA$, and compared between images rendered with blur (*COMP*) and images blurred dioptrically for + 1, + 2 or + 4 D of blur. We additionally tested the importance of compensating for the short viewing distance and the vertex distance—the shortest distance between the cornea and lens—by including dioptric blur conditions with (*OPTadj*) and without this correction (*OPT*).

## Results

**Figure 1:**
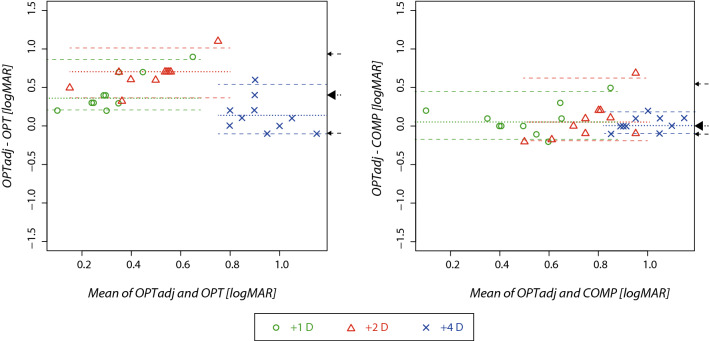
Bland–Altman^15^ plot compares measured VA between the *OPT* and *OPTadj* methods (left panel) and the *COMP* and *OPTadj* methods (right panel). Along the horizontal axis, the mean, and along the vertical axis, the difference of two VA measurements are plotted. Every point corresponds to a single participant and the symbols and colors differentiate the blur levels. The horizontal dotted lines represent the median difference between the two compared methods and the dashed lines indicate the limits of agreement (2.5–97.5 quantile range). The medians and limits of agreement are depicted separately for the particular level of blur, the arrows on the right side of plot indicate the median and limits of agreement for all values irrespective of the blur level. Results of dioptric blur using a direct addition of + 1 and + 2 D lenses (*OPT* method) were biased toward a higher VA (lower logMAR). The rendered blur approach (*COMP* method) was not statistically different from the adjusted dioptric approach (*OPTadj* method)—see Table 2.

The VA measured in 10 male subjects (mean age 35 years) using all three methods (*COMP*, *OPT* and *OPTadj*) are listed in Table 1 for all levels of blur. For all tested levels of defocus, the VA values measured with the *OPTadj* method were not statistically different from those measured using the *COMP* method (z = 1.3 p = 0.57). This was confirmed for each level of defocus (p > 0.20) using paired tests. The median intra-individual difference with interquartile range in VA (*OPTadj − COMP*) was 0.05 (− 0.18, 0.17) logMAR for + 1 D, 0.05 (− 0.20, 0.18) logMAR for + 2 D and 0.00 (− 0.10, 0.10) logMAR for + 4 D. The median intra-individual difference with interquartile range in VA (*OPTadj* − *OPT*) was 0.35 (0.20, 0.62), 0.70 (0.60, 0.70) and 0.10 (− 0.1, 0.2) logMAR for + 1, + 2 and + 4 D respectively. This demonstrates that the VA values measured by the *OPT* method were significantly lower for + 1 D (p = 0.006) and + 2 D (p = 0.006) blur than for the *OPTadj* method, however, for + 4 D the statistical significance was not confirmed (p = 0.07). The relationship is depicted in Fig. 1, which illustrates the intra-individual differences in VA and the effect of the level of defocus in a Bland–Altman^15^ plot. The limits of agreement (i.e. the interval from the 2.5 to the 97.5 quantile—see “Methods”) for the *OPTadj*–*COMP* methods were − 0.18 to 0.45 logMAR for + 1D, − 0.20 to 0.59 logMAR, for + 2D and − 0.10 to 0.18 logMAR for + 4 D. When comparing the *OPT* to the *OPTadj* method the limits of agreement were 0.20–0.85 logMAR for + 1D, 0.36–1.01 logMAR for + 2D and − 0.10 to 0.55 log MAR for + 4D. All limits of agreements are listed in Table 2. The width of limits of agreement for *OPTadj*–*OPT* methods was always 0.65 logMAR. The corresponding widths of the limits of agreement between *OPTadj*–*COMP* were 0.63, 0.79 and 0.28 logMAR for + 1, + 2 and + 4 D, respectively.

**Table 1:** VA (logMAR) and descriptive statistics for all three methods and levels of the blur; 25% (75%)—lower (upper) quartile; S01-S10—participant's code.

Participant	logMAR—digital defocus (*COMP*)				logMAR—dioptric defocus (*OPT*)			logMAR—adjusted dioptric defocus (*OPTadj*)			
	0 D	1 D	2 D	4 D	1 D	2 D	4 D	1 D	2 D	4 D	4 D—novertex c
S01	0.2	0.6	1.0	1.1	0.2	0.2	1.0	0.5	0.9	1.0	1.0
S02	0.2	0.6	0.6	0.9	0.2	0.2	1.2	1.1	1.3	1.1	1.3
S03	0.2	0.7	0.8	0.9	0.1	0.2	0.8	0.5	0.9	0.8	1.1
S04	0.2	0.4	0.8	0.9	0.1	0.1	0.8	0.4	0.7	0.9	1.0
S05	-0.1	0.0	0.6	0.9	0.0	-0.1	0.7	0.2	0.4	0.9	0.9
S06	0.2	0.5	0.7	1.0	0.1	0.2	0.7	0.5	0.9	1.1	1.1
S07	0.2	0.5	0.7	1.1	0.1	0.2	1.0	0.8	0.9	1.1	1.3
S08	0.2	0.3	0.7	1.1	0.2	0.2	0.6	0.4	0.5	1.2	1.1
S09	0.2	0.4	0.7	0.9	0.1	0.0	1.0	0.4	0.7	0.9	1.0
S10	0.1	0.6	0.7	0.9	0.0	0.2	0.8	0.7	0.8	1.0	1.2
Median	0.20	0.49	0.70	0.90	0.10	0.20	0.80	0.49	0.85	1.00	1.10
25%	0.20	0.40	0.70	0.90	0.10	0.12	0.72	0.40	0.70	0.90	1.00
75%	0.20	0.60	0.77	1.07	0.17	0.20	1.00	0.65	0.90	1.10	1.18

The last column represents VA measured for OPTadj condition without the vertex correction.

**Table 2:** VA (logMAR) comparisons between different methods of blur.

Defocus level	*OPTadj—COMP *median (limits of agreement) [logMAR]	*OPTadj—COMP *comparison p value	*OPTadj—OPT *median (limits of agreement) [logMAR]	*OPTadj—OPT*comparison p value
1 D	0.05 (− 0.18 0.45)	0.2038	0.35 (0.20 0.85)	0.0059*
2 D	0.05 (− 0.20 0.59)	0.5933*	0.70 (0.36 1.01)	0.0056*
4 D	0.00 (− 0.10 0.18)	0.4431*	0.1 (− 0.10 0.55)	0.0748

A median closer to zero and narrower limits of agreement indicate better agreement between compared methods. The p value relates to the result of a paired Student t test or a Wilcoxon sign test (*).

## Discussion

In our experiment we simulated blurred vision using source image modification by rendering stimuli with added Zernike aberrations (*COMP*) to test if a low-resolution display (65 dpi) observed from 60 cm can reliably simulate defocus for recordings that require a short viewing distance, such as visual evoked potentials and pattern electroretinograms. We compared this approach with dioptric defocus created by placing lenses in front of the eye. The combined power of the lenses was adjusted for a short observing distance and vertex distance (*OPTadj*). Additionally, we recorded VA to a naïvely uncorrected dioptric defocus (*OPT*). This recording we used to demonstrate the importance of the corrections used in the *OPTadj* method and to verify the sensitivity of our statistical approach.

Although the median VAs measured for the *COMP* method for + 1 D and + 2 D of defocus were marginally better (0.05 log MAR) than for the *OPTadj* method (i.e., the *COMP* method produced slightly less blurred images than the *OPTadj* method), we did not find a statistically significant difference between these two methods. This is in broad agreement with the studies of Smith et al*.*^8^, Ohlendorf et al*.*^9^, Remón et al*.*^10^ and Dehnert et al*.*^11^ in that simulated and dioptric defocus result in comparable VA measurements. Furthermore, our results also show a slight tendency to measure a lower VA with digital blur, compared to dioptric blur, which is in agreement with Dehnert et al.^11^, but contrary to the other studies^8–10^. The biggest median difference measured was − 0.05 logMAR (interquartile range from − 0.18 to 0.10 logMAR) for + 2 D, but this is not ecologically and clinically important; a greater difference of 0.13 ± 0.04 (mean ± SEM) logMAR for + 2 D blur was considered in the study of Dehnert et al.^11^ as a reasonable compromise in exchange for the advantages of computer simulation. Furthermore, the median intra-individual change in VA between the *OPTadj* and *COMP* methods corresponds to only half a line of the Early Treatment Diabetic Retinopathy Study (ETDRS) chart^16^.

Another benefit of including the *OPT* method into our experimental design was ability to evaluate test–retest limits of agreement using Bland Altman analysis. Since the width of the limits of agreement depends on measurement variability only, the different magnification power between the *OPTadj* method and the *OPT* method does not influence it and we can use this width as a reference for assessment of the variability between the *OPTadj–COMP* methods. The *OPTadj-COMP* limits of agreement widths for + 1 D and + 4 D were narrower than, and for + 2D close to, that for the *OPTadj–OPT* method. The limits of agreement between the *OPTadj* and *COMP* methods for + 4 D blur (− 0.10 to 0.18 logMAR) corresponds to limits for test–retest examination in the clinical population (± 0.16 logMAR)^17^. These results suggest that measurement variability when comparing the COMP and OPT*adj* methods was similar or better than when comparing the two purely optical methods, see Table 2.

In our experiment, we have used an estimated pupil diameter of 5 mm to calculate the RMS wavefront error in the rendering our stimuli. The reason why we have not used a measurement of observers’ pupil diameter is that the pupil size changes between individuals and over time with cognitive load or fatigue^18^. It would be impractical to measure the pupil diameter and perform the stimulus rendering before the experiment and computationally challenging to update the display in real-time as the pupil changes in size. Our results show that using the estimated pupil size resulted in a measure of VA that did not deviate significantly from that measured using dioptric blur. However, an overestimation of the pupil diameter may explain the slightly better VA measured with the *COMP* method than the *OPTadj* method.

**Figure 2:**
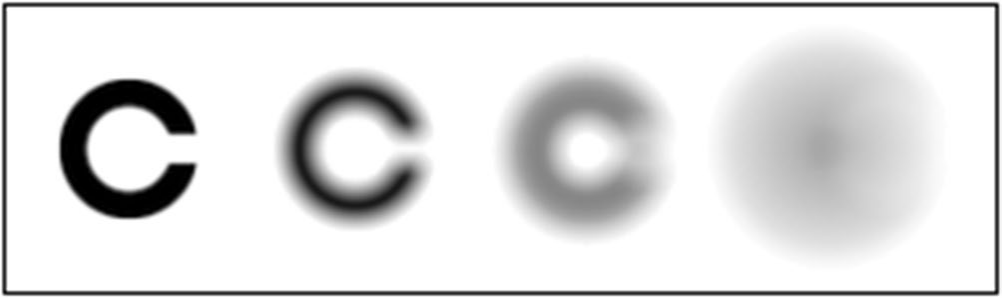
Landolt Rings rendered with 0, 1, 2 and 4 D of equivalent defocus. The angular size of Landolt Ring was 100’.

One should consider that spurious resolution, as a consequence of the optical/modulation transfer function shape^19^, might affect the VA for higher levels of blur. Figure 2 shows, at the highest level of equivalent defocus, an effect of spurious resolution—there is a contrast reversal between the centre and the annulus of the Landolt Ring. Our results suggest that, as a close match is maintained across all levels of defocus (see Fig. 1), it is unlikely that the *COMP* and *OPTadj* methods differed in the spurious resolution presented to the participant, and so spurious resolution likely did not lead to a difference between the methods.

As expected, we found that it was important to correct for viewing distance and vertex distance in the dioptric approach. This highlights another benefit of the *COMP* approach, which does not have this requirement, and those results showed lower variability at higher levels of defocus than what we achieved dioptrically (see Fig. 1). Considering the possibility that the eye lid can change the effective size and shape of the pupil, further influencing the optical defocus, our results support the rendering method as a robust approach to simulate spherical refractive error.

In our experiment we also evaluated whether dioptric defocus approximated by the simple use of a lens of the desired power (*OPT* method) produces significantly different results to dioptric defocus using lenses adjusted for the observation and vertex distances (*OPTadj* method). At lower blur levels, measured VA with Landolt Rings defocused via the *OPT* method were significantly better than those measured via the *OPTadj* method. This was likely due to the short viewing distance—the accommodative demand for a 60 cm viewing distance is + 1.67 D and so the refractive error introduced by a + 1 D or even a + 2 D lens could be overcome by the remaining accommodative capacity of the eye. This was also demonstrated by an overlap in measured VA for + 1 D and + 2 D for the *OPT* method (indicating that they produced a similar amount of blur on the retina) and no difference to the control condition (+ 0 D COMP, see Table 1). From these results we conclude that, in a short observing distance, omitting a correction for observation and vertex distances had a behaviourally important effect and so this correction should be performed.

Additionally, we verified the importance of the vertex distance correction. For the highest level of defocus (+ 4 D) and a vertex distance of 12 mm, the power of the eye–lens system increased to + 4.2 D. This already resulted in a statistically significant difference (p < 0.034) between the accommodation-corrected + 4 D condition (last column in Table 1) and the *OPTadj* approach, in which accommodation and vertex were corrected for. The effect size was 1.03 (0.03–2.3 95% confidence interval) and the median intra-individual VA decrease was − 0.10 logMAR.

For all participants, the measured VA for non-blurred Landolt Rings without accommodation correction were on average 0.16 logMAR (minimal angle resolution of the eye of 1.45′), which is worse than the worst VA of the standard observer (visual angle of 1.25′, i.e. 0.1 logMAR)^20^. Considering a one-pixel wide gap (visual angle of 2.23′) in the Landolt Ring, we would expect a VA of 0.35 logMAR. The measured VA was therefore better than one would expect based on the resolution of the monitor. Such a VA enhancement was most likely due to an unbalanced spatial distribution of light across the Landolt Ring, which facilitated the Landolt Ring gap detection^21^. The unbalanced light distribution was caused by image undersampling resulting in a lower, but still detectable, contrast across the Landolt Ring gap.

## Conclusion

Our results showed that VA measured using digitally blurred images by adding Zernike defocus in their rendering was not statistically different from dioptric defocus using trial lenses and correcting for the observation and vertex distances. For the higher level of defocus, 4 D in our case, these methods were interchangeable. Our data were acquired with a short observing distance and on a low-resolution display, while presenting Landolt Rings with + 1, + 2 or + 4 D of equivalent defocus. Using computer rendered stimuli represents an easier and robust approach to testing refractive errors when a short observation distance is required. We provide a set of digitally blurred Landolt Rings that allow replication of our experiment. All materials are published in the appendix hereto.

## Methods

### Background

Wavefront aberrations, $W\left(r,\theta \right)$, such as spherical refractive error can be quantified in terms of their Zernike coefficients^22^ according to the Zernike polynomial expansion^23,24^,

1 $$W\left(r,\theta \right)=W\left(R\rho ,\theta \right)=\sum_{n=0}^{\infty }\sum_{m=-n}^{n}{C}_{n}^{m}\cdot {Z}_{n}^{m}\left(\rho ,\theta \right).$$

The terms ${Z}_{n}^{m}$ are Zernike polynomials and the terms ${C}_{n}^{m}$ represent the corresponding Zernike coefficients. The lower index $n$ in each expansion term indicates the radial order of the aberration and the upper index $m$ denotes the angular order. For any given $n\in {\mathbb{N}}$, $n\ge m$ and $n-m$ must be even, and the index *m* takes values from $-n \mathrm{to} n$ in steps of 2. The variable $\theta$ is the azimuthal angle and $\rho =r/R$ is the normalized radial position, where *r* is the radial coordinate and R is the pupil radius. Spherical refractive errors can be approximated as the second order aberration $(n=2)$ defocus, represented by Zernike polynomial ${Z}_{2}^{0}$, which is described in polar coordinates by^25^:

2 $${Z}_{2}^{0}\left(\rho ,\theta \right)=\sqrt{3}\cdot \left(2\cdot {\rho }^{2}-1\right).$$

The point spread function (*PSF*) of an optical system is given by^23^,

3 $$PSF\left(r,\theta \right)={\left|\text{F}\left\{P\left(r,\theta \right)\right\}\right|}^{2}={\left|\text{F}\left\{p\left(r,\theta \right)\cdot exp\left(i\cdot \frac{2\pi }{\lambda }\cdot W\left(r,\theta \right)\right)\right\}\right|}^{2}.$$

The complex aperture function, $P\left(r,\theta \right),$ is composed of a real component, the transmission function, $p\left(r,\theta \right),$ as well as the complex wavefront, $W\left(r,\theta \right)$. We approximate the transmission function by a top-hat function with 0 outside the circular pupil and 1 within the circular pupil, rather than approximating the Stiles-Crawford effect^11^. The PSF for our selected defocus aberration has the following form,

4 $$PSF\left(r,\theta \right)={\left|\text{F}\left\{p\left(r,\theta \right)\cdot exp\left(i\cdot \frac{2\pi }{\lambda }\cdot {C}_{2}^{0}\cdot \sqrt{3}\cdot \left(2\cdot {\rho }^{2}-1\right)\right)\right\}\right|}^{2},$$

where ${C}_{2}^{0}$ is the RMS Zernike coefficient for defocus. The resulting intensity pattern $I^\prime\left(r,\theta \right)$ representing an image observed through an optical system with spherical refractive error, i.e., a defocus aberration, is given by the convolution of the input intensity pattern $I\left(r,\theta \right)$ and the PSF.

The PSF pixel scale (${s}_{PSF}$) and the pixel scale of the source image (${s}_{obj}$) must be the same, i.e., ${s}_{PSF}={s}_{obj}$, for correct construction of the transformed image. We employed Fraunhofer's diffraction theory for a rectangular aperture (the shape of the image array) to determine the pixel scale of the PSF function as^26^ ${s}_{PSF}=\frac{\lambda }{D\cdot \alpha }$, where $\lambda$ is the wavelength of the light used, $D$ is the diameter of the aperture and $\alpha$ is the oversampling factor^24^. We use $\alpha =2$ in our study. The source image pixel size is expressed as ${s}_{obj}=\frac{{v}_{obj}}{{N}_{obj}}$, where ${N}_{obj}$ is the number of pixels across the field of view and ${v}_{obj}$ is the field of view of the source image. Since the pixel scales of the PSF and image must match, $\frac{{v}_{obj}}{{N}_{obj}}=\frac{\lambda }{D\cdot \alpha }$, and hence,

5 $${N}_{OBJ}=\frac{{v}_{OBJ}\cdot D\cdot \alpha }{\lambda }$$

### Stimuli

Some experiment settings, like the 60 cm observing distance, were defined with respect to future utilisation of digital blur in visually evoked potentials studies. Stimuli were displayed on a CRT monitor (Mitsubishi Diamond Pro 2070 SB, Japan) with a resolution of $1024\times 768 \mathrm{pixels}$ and a display area of $40\times 30 \mathrm{cm}$ (i.e. 65 dpi). A viewing distance of 60 cm resulted in a pixel size of 2.23′. The average luminance of the stimulus was kept at 17 cd/m^2^ and participants were examined in a darkened cabin with a background luminance of 1 cd/m^2^.

Based on^27^, we created 14 Landolt Rings of angular size of 5′, 6.25′, 8′, 10′, 12.5′, 16′, 20′, 25′, 31.5′, 40′, 50′, 62.5′, 80′ and 100′. Each ring was blurred digitally by convolving it with the PSF described in Eq. (4). Stimuli were generated using custom code written in the Python programming language using the NumPy library^28^ and blur was added during the rendering^24^. A complete set of generated rings is available in the Supplementary Material S1.

We chose to simulate myopia instead of hypermetropia because participants cannot overcome the induced positive spherical error by accommodating, whereas for a negative spherical error (hypermetropia), they could accommodate through the lens and reduce the blur. We used equivalent defocus values of + 1, + 2 and + 4 D—example stimuli are given in Fig. 2. The relationship between the RMS Zernike defocus coefficient, ${C}_{2}^{0},$ the equivalent defocus, *M*_*e*_, and the pupil area, *A,* is^29^:

6 $${M}_{e}=4\cdot \pi \cdot \sqrt{3}\cdot \frac{{C}_{2}^{0}}{A}=\frac{4\cdot \sqrt{3}\cdot {C}_{2}^{0}}{{R}^{2}}.$$

We assumed an average the pupil diameter of 5 mm (in a dark room with low average luminance stimuli) and so these values correspond to RMS values of 0.9, 1.8 and 3.6 μm, and these were kept constant across participants.

### Examination

We tested 10 male participants aged between 20 and 49. The study was approved by the Ethical Committee of University Hospital in Hradec Kralove, all tenets of the Declaration of Helsinki were followed, and all participants provided informed consent.

For the examination, we selected the eye whose best corrected refractive state, measured with an autorefractometer (Full Auto Ref R-F10, Canon, Ltd., Japan), corresponded more closely to the emmetropic eye, or in case of equality we selected the dominant eye determined by the hole-in-the-card test.

The blur was introduced using three methods: (1) *OPT* (dioptric blur)—a sharp Landolt Ring viewed through gradually added external positive lenses placed in front of the participant’s eye; (2) *OPTadj*—as for *OPT* but with a correction described below; and (3) *COMP*—digitally-blurred Landolt Ring utilising the method described above.

When examining VA the eye is not fully accommodated to infinity, so a lens with a refractive power of 0.167 D should be placed in front of the eye for 6 m examination^20^. In our study, with an observing distance of 60 cm, we used an external lens with the refractive power of 1.67 D for the *OPTadj* method.

Together, the lens and the eye constitute an optical system, the focal properties of which depend on the vertex distance measured along the optical axis between the cornea and the external lens. For our participants, an average vertex distance of 12 mm resulted in a negligible lens adjustment to achieve the desired refraction error of + 1 or + 2 D. In the case of + 4 D, the optical system power adjustment is already 0.2 D, therefore we adopted + 3.8 D instead of 4 D for the *OPTadj* method. The placement of a lens in front of the eye’s entrance pupil also results in a magnification of the retinal image, with a magnification factor of $M=\frac{1}{1-a{F}_{SPH}}$, where *F*_*SPH*_ is the power of the lens and *a* is the distance from the lens to the entrance pupil of the eye^30^. We assume *a* to be the vertex distance plus 3 mm, to account for the distance between the entrance pupil and the corneal surface^30^. For the 12 mm vertex distance used in this study, the magnification factors associated with each of the lenses tested and the equivalent change in logMAR were as follows: for the OPT method − 1.015 and 0.007 logMAR for the 1 D lens, 1.031 and 0.013 logMAR for the 2 D lens, and 1.064 and 0.027 logMAR for the 4 D lens; for the OPT*adj,* with an additional 1.67 D lens − 1.042 and 0.018 logMAR for the 1 D condition, 1.058 and 0.025 logMAR for the 2 D condition, and 1.089 and 0.037 logMAR for the 4 D (3.8 D) condition. Even the largest magnification difference is well below 0.1 logMAR and is therefore not considered to produce a clinically significant change in VA.

For all three methods, the VA examination was performed using a Java application developed for this project, which displayed a single Landolt Ring in the middle of a monitor. For each angular size, the Landolt Ring was presented 10 times in an orientation pseudo-randomly rotated by 0°, 90°, 180°, or 270°. Subjects indicated the position of the gap in the Landolt Ring by pressing the corresponding arrow key on a computer keyboard. VA for each of the three methods was determined by the angular size of the Landolt Ring gap for which the number of errors (number of misidentified gap positions) was less than or equal to four^27^. The Landolt Ring presentation for each method began from the smallest to the largest ring. We intentionally used the routine clinical method instead of measuring the psychometric function.

For all three methods, we used meniscus trial lenses (Art. 51-BL, M.S.D., Italy) with spherical correction lenses in + 0.12, + 0.25, 0.5, + 0.75, + 1.0, + 1.25, + 1.5 D, etc. steps. For the *COMP* method, we used a combination of trial lenses during the examination such that the refractive state of the investigated participant's eye was closest to a refractive error of 0 D. For the *OPT* and *OPTadj* methods, we aimed for a combination of lenses such that the refractive power was as close as possible to the subject’s refraction prescription measured by a refractometer, and then added + 1 D, + 2 D, or + 4 D for the *OPT* method or + 2.67 D, + 3.67 D, or + 5.47 D for the *OPTadj* method. The difference between such theoretical value and the value obtained by the combination of trial lenses positioned in front of the subject’s eye was at most 0.01 D (in two cases) for the *OPT* method and 0.04 D in three participants and 0.05 D in the rest of the group for the *OPTadj* method.

### Statistical analyses

To test differences among defocus methods we adopted a two-step analysis. In the first step we checked whether the mean or median (dependent on the data distribution) of VA in logMAR differs significantly between *OPTadj* and *COMP* method, and between *OPTadj* and *OPT* method. Based on data distribution assessed by the Anderson–Darling test we used either a Wilcoxon signed rank test or a paired Student’s t-test. A correction for multiple comparisons was not made because it would lower the significance level, increasing the likelihood of a Type 2 error. As this study assesses the suitability of simulated defocus as an alternative to optical defocus, a Type 2 error is of most concern as it could lead us to conclude that the two methods were equivalent even if they were not.

In the second step, we used the Bland–Altman approach to evaluate an agreement between the aforementioned pairs of defocus methods. Beside the judgement of the mean or median values of particular methods, which we covered in the first step, Bland–Altman analysis computes the 95% limits of agreement (the range from the 2.5 to the 97.5 quantile) using paired differences between appropriate methods. We compared the limits of agreement for *OPTadj*–*COMP* differences to the limits of agreement for *OPTadj*–*OPT* differences, and to data in the literature. As an important part of the Bland–Altman analysis we also depicted differences between paired data against their averages.

Because part of our data did not follow normal distribution, we used the median and quantiles as summary statistics, instead of the mean and the standard deviation. Statistical analysis was conducted in *R*^31^ using the *BlandAltmanLeh* package^32^ and the level of statistical significance, alpha, was 5%.

## Ethics approval

All procedures performed in our study were in accordance with the ethical standards of the institutional and/or national research committee and with the 1964 Helsinki declaration and its later amendments or comparable ethical standards. The study was approved by the Ethical committee of University Hospital in Hradec Kralove (no. 201411519P).

## Supplementary Information

Supplementary Information 1.

Supplementary Information 2.

## Data Availability

The datasets generated during and/or analyzed during the current study are partially available as supplementary material to this article, the rest is obtainable from the corresponding author on reasonable request.

## Code availability

The Python code for blur rendering is available elsewhere (http://sourceforge.net/projects/aberrationrendering/), the analysis script is available from the corresponding author on reasonable request.
