# Supplementary material for: Comparison between optical and digital blur using near visual acuity
Source: Sci Rep. 2021 Feb 9;11:3437. doi: 10.1038/s41598-021-82965-z (PMC7873285; doi:10.1038/s41598-021-82965-z)
Supplement: Supplementary file 1 — Supplementary Information 1. [file 41598_2021_82965_MOESM1_ESM.docx]

**Comparison between optical and digital blur using near visual acuity**

Kordek David^a^, Young Laura K.^b,c^, Kremláček Jan^a,d,*^

^a^Department of Medical Biophysics, Charles University - Faculty of Medicine in Hradec Kralove, Czech Republic

^b^ Biosciences Institute, Faculty of Medical Sciences, Newcastle University, Newcastle, UK

^c^Department of Experimental Psychology, University of Oxford, Oxford, UK

^d^Department of Pathological Physiology, Charles University in Prague - Faculty of Medicine in Hradec Kralove, Czech Republic

***Corresponding author**

Jan Kremláček, Faculty of Medicine in Hradec Kralove, Simkova 870, 500 38 Hradec Kralove, Czech Republic, e-mail: jan.kremlacek@lfhk.cuni.cz, tel: 420 495 816 332

**Supplementary materials**

**A** – Python code for the *COMP* method has been published elsewhere (http://sourceforge.net/projects/aberrationrendering/)^24^

**B** – A set of Landolt Rings rendered with an equivalent defocus of +1, +2 or +4 D using the *COMP* method. To keep the designed angular size of the Landolt Ring, the whole image (768×768 pixels) should subtend 28×28° of visual angle, giving an angular size of one pixel of $2.23´\times2.23$´. In *digitally_blured_Landolt_rings.zip* archive, there are the images sorted into four directories (0D, 1D, 2D and 4D) corresponding to level of defocus. Individual file names denote the size of the Landolt Ring, i.e. file *100_00.BMP* contains Landolt Ring with an outer diameter of 100.00’.

**C** – The optical power of lenses used in the experiment

**Table S3.** Dioptric values of the theoretical lenses required (Ideal), the pre-positioned combination of lenses tested (Used), and their differences for all 10 subjects. The theoretical value was determined as the sum of the optical blur required and the measured refractive power of an observer’s eye. For each observer, the dioptric power that was used to produce 0 D of blur was used for all other blur levels when testing the COMP method. The optical powers for the OPT and OPTadj methods are listed just for 1 D blur, but the Ideal-Used differences were the same for 2 D and 4 D blur.

| *Optical power used* | *0 D*  *(COMP)* | | | *1 D*  *(OPT)* | | | *1+ 1.67 D*  *(OPTadj)* | | |
| --- | --- | --- | --- | --- | --- | --- | --- | --- | --- |
| *Subject* | *Ideal*  *[D]* | *Used*  *[D]* | *Ideal -Used [D]* | *Ideal*  *[D]* | *Used*  *[D]* | *Ideal -Used [D]* | *Ideal*  *[D]* | *Used*  *[D]* | *Ideal -Used [D]* |
| S01 | -0.25 | -0.25 | 0.00 | 0.75 | 0.75 | 0.00 | 2.42 | 2.37 | 0.05 |
| S02 | -0.25 | -0.25 | 0.00 | 0.75 | 0.75 | 0.00 | 2.42 | 2.37 | 0.05 |
| S03 | 0.25 | 0.25 | 0.00 | 1.25 | 1.25 | 0.00 | 2.92 | 2.87 | 0.05 |
| S04 | 0.12 | 0.12 | 0.00 | 1.12 | 1.12 | 0.00 | 2.79 | 2.75 | 0.04 |
| S05 | -0.25 | -0.25 | 0.00 | 0.75 | 0.75 | 0.00 | 2.42 | 2.37 | 0.05 |
| S06 | 0.00 | 0.00 | 0.00 | 1.00 | 1.00 | 0.00 | 2.67 | 2.62 | 0.05 |
| S07 | -0.25 | -0.25 | 0.00 | 0.75 | 0.75 | 0.00 | 2.42 | 2.37 | 0.05 |
| S08 | 0.37 | 0.37 | 0.00 | 1.37 | 1.37 | 0.00 | 3.04 | 3.00 | 0.04 |
| S09 | -0.50 | -0.50 | 0.00 | 0.50 | 0.50 | 0.00 | 2.17 | 2.12 | 0.05 |
| S10 | -0.25 | -0.25 | 0.00 | 0.75 | 0.75 | 0.00 | 2.42 | 2.37 | 0.05 |
| *Mean* | -0.10 | -0.10 | 0.00 | 0.90 | 0.90 | 0.00 | 2.57 | 2.52 | 0.05 |
| *S.D.* | 0.27 | 0.27 | 0.00 | 0.27 | 0.27 | 0.00 | 0.27 | 0.28 | 0.00 |
