# Supplementary material for: Comparison between optical and digital blur using near visual acuity
Source: Sci Rep. 2021 Feb 9;11:3437. doi: 10.1038/s41598-021-82965-z (PMC7873285; doi:10.1038/s41598-021-82965-z)
Supplement: Supplementary file 2 — Supplementary Information 2. [file 41598_2021_82965_MOESM2_ESM.pdf]

Nejlepších výsledků dosáhnete, když toto portfolio PDF otevřete v aplikaci Acrobat X či Adobe Reader X nebo novější.

Opatřete si Adobe Reader nyní!
